# Nitrogen supply modulates carbon–nitrogen balance and metabolic profiles in contrasting onion varieties

**DOI:** 10.3389/fpls.2026.1886540

**Published:** 2026-08-14

**Authors:** M. L. Romo-Pérez, C. H. Weinert, B. Egert, S. E. Kulling, C. Zörb

**Affiliations:** 1 Quality of Plant Products 340e, Institute of Crop Science, University of Hohenheim, Stuttgart, Germany; 2 Institute for Foods of Plant Origin, Max Rubner-Institut, Karlsruhe, Germany; 3 Department of Safety and Quality of Fruit and Vegetables, Max Rubner-Institut, Karlsruhe, Germany

**Keywords:** *Allium cepa L.*, carbon, hybrids, landraces, metabolomics, nitrogen, quality

## Abstract

Onion bulb quality is largely defined by non-structural carbohydrates, phenolic antioxidants, and sulfur-containing flavor precursors, yet integrative evidence on how these domains respond to nitrogen imbalance under controlled conditions remains limited. A pot experiment applied four ammonium nitrate levels (N1–N4; 0.5–4.0 g NH_4_NO_3_ per pot) to two contrasting onion varieties (hybrid Hytech F1 and landrace Birnenförmige). Bulb morphology and quality traits were quantified alongside tissue elemental C:N ratios and a targeted trait–metabolite matrix covering carbohydrates, amino acids (including Allium-specific alk(en)yl cysteine sulfoxides, ACSOs), organic acids and other primary metabolites, as well as flavonoids, driven by higher dry matter and fructan-rich reserve pools in Birnenförmige versus higher soluble sugars and nitrogen-status metabolites in Hytech. Along the nitrogen gradient, bulb growth followed a non-linear response, peaking at intermediate supply and declining under oversupply, with a stronger high-N penalty in Hytech. Nitrogen oversupply increased nitrate and free amino acid pools and lowered elemental bulb C:N ratios, while total phenolic content and quercetin glycoside levels declined. In contrast, relative levels of putatively annotated ACSO-like compounds increased as pyruvate-based pungency decreased, consistent with a compositional decoupling between sulfur-containing compound profiles and the pyruvate-based pungency signal under high N. Together, the results identify variety-specific thresholds and coordinated trade-offs linking nitrogen status to growth and the major quality-related metabolic domains in onion bulbs.

## 1 Introduction

Onion (*Allium cepa* L.) is among the most widely produced vegetable crops worldwide (FAO, 2023), and bulb size and composition are central targets in onion production and processing (Kinoshita et al., 2024). Nitrogen management is a central lever in vegetable production because it can shape both biomass formation and edible quality traits. In bulb onion, nitrogen supply often increases vegetative growth and bulb size up to an intermediate range, yet responses can plateau or even reverse under high inputs, particularly where nutrient supply can exceed plant sink capacity (Randle, 2000; Geisseler et al., 2022). Beyond yield responses, nitrogen availability can reprogram primary metabolism by increasing nitrogen-rich soluble pools such as nitrate and free amino acids, reflecting both assimilation activity and storage buffering when nitrogen supply exceeds immediate growth demand (Lawlor, 2002; Masclaux-Daubresse et al., 2010). Such shifts affect internal carbon–nitrogen balance and may be associated with changes in elemental tissue composition, non-structural carbohydrate pools, and quality-related metabolism.

Onion bulb quality is largely shaped by composition, with three metabolic groups being particularly relevant: non-structural carbohydrates, antioxidant flavonoids (especially quercetin derivatives), and sulfur-containing flavor precursors. A large fraction of bulb dry matter consists of non-structural carbohydrates, including fructans and soluble sugars, which contribute to sweetness, texture, processing properties, and storage behavior (Griffiths et al., 2002; Petropoulos et al., 2017). Onion bulbs are also a major dietary source of flavonoid antioxidants, and quercetin derivatives are among the most characteristic compounds; their accumulation can be sensitive to carbon status and nitrogen supply (Griffiths et al., 2002; Mogren et al., 2006; Stefanelli et al., 2010). Finally, characteristic onion flavor and pungency arise from sulfur-containing alk(en)yl cysteine sulfoxides (ACSOs) and their conversion into reactive products after tissue disruption. Pyruvic acid, generated by alliinase-catalyzed degradation of ACSOs upon tissue disruption, is widely used as a practical pungency proxy, but it reflects an enzymatically developed signal that depends on precursor pools and post-disruption conversion (Randle, 2000; Kato et al., 2016). Together, these three metabolic domains define much of the sensory and nutritional value of onion bulbs, yet they may respond to nitrogen supply in different, and sometimes opposing, directions.

A further layer of complexity is that genotype can dominate bulb composition and nitrogen responsiveness. Onion varieties can differ strongly in carbohydrate partitioning (reserve fructans *vs*. soluble sugars) and in baseline levels of nitrogen-related metabolites and secondary compounds (Romo-Pérez et al., 2026), which can shape how quickly a variety responds across an N gradient. Metabolomics work in onion highlights broad cultivar-dependent differences across sugars/fructans, amino acids, and sulfur-containing metabolites, underlining that “quality” is not a single trait but a coordinated phenotype (Saviano et al., 2019; Romo-Perez et al., 2020). Beyond onion, comparative studies in other crops suggest that landraces often show flatter yield responses across nitrogen levels than modern hybrids, consistent with greater yield stability under N limitation but lower responsiveness under high N (Ferro et al., 2006; Gaju et al., 2016). These patterns motivate explicit variety contrasts when interpreting nitrogen effects on both growth and composition.

Despite the agronomic importance of nitrogen for onion, integrative controlled studies that track nitrogen-driven shifts across the major quality-related metabolic groups, carbohydrates, phenolic antioxidants, and sulfur-containing compounds remain limited. Recent onion nitrogen work is frequently field-based and focuses on agronomic N ranges, where strong under- and oversupply contrasts are difficult to establish and mechanistic interpretation is complicated by environmental variation (Golubkina et al., 2022; Yeshiwas et al., 2024). The present study therefore uses a controlled pot nitrogen gradient combined with a broad trait–metabolite matrix to quantify (*i)* the relative importance of variety versus nitrogen supply, (*ii*) growth and nitrogen-status responses across the nitrogen gradient, including an oversupply regime, and (*iii*) coordinated shifts in bulb composition across the three main quality-related metabolite groups: carbohydrates, phenolic antioxidants, and sulfur/pungency compounds.

To address these gaps, a controlled pot experiment was conducted with two contrasting onion varieties (a hybrid F1 and a landrace variety) grown under a graded nitrogen supply spanning deficiency to oversupply (four N levels). Bulb growth and structural traits were quantified alongside mineral N and elemental C:N status, and quality-related composition was assessed across carbohydrates, phenolic antioxidants, and sulfur/pungency traits. In addition, a broad metabolite dataset was used to capture coordinated shifts in primary and secondary metabolism along the nitrogen gradient.

## 2 Materials and methods

### 2.1 Plant material and growth conditions

The experiment was carried out in spring-summer 2022 at the experimental facilities of the University of Hohenheim (48°42′29.149” N, 9°12′42.25” E). Seeds of the landrace variety Birnenförmige (Sativa Biosaatgut GmbH, Germany) and the hybrid variety Hytech F1 (Bejo Seeds, The Netherlands) were sown in trays and grown for six weeks under controlled conditions (day/night temperature 25 °C/18 °C, natural light without artificial illumination, and automatic ventilation). During seedling establishment, plants were exposed to the natural photoperiod at the greenhouse location, with daylength increasing from approximately 12.1 to 14.5 h. After establishment, seedlings were transplanted into Mitscherlich pots (6.02 kg soil per pot) filled with a 1:1 mixture of loam and sand and grown in a net-covered outdoor growing area under ambient seasonal conditions. During the main growth period from May to August 2022, natural daylength ranged from approximately 14.4 to 16.2 h. Based on weather-station data from the University of Hohenheim, monthly mean air temperature ranged from 15.4 °C in May to 21.1 °C in August, with recorded monthly extremes between 3.4 and 35.4 °C. Each pot contained four plants. Soil pH was adjusted to 7.0 by incorporating 5% (w/w) sour turf prior to planting.

### 2.2 Nitrogen treatments

**Figure 1:**
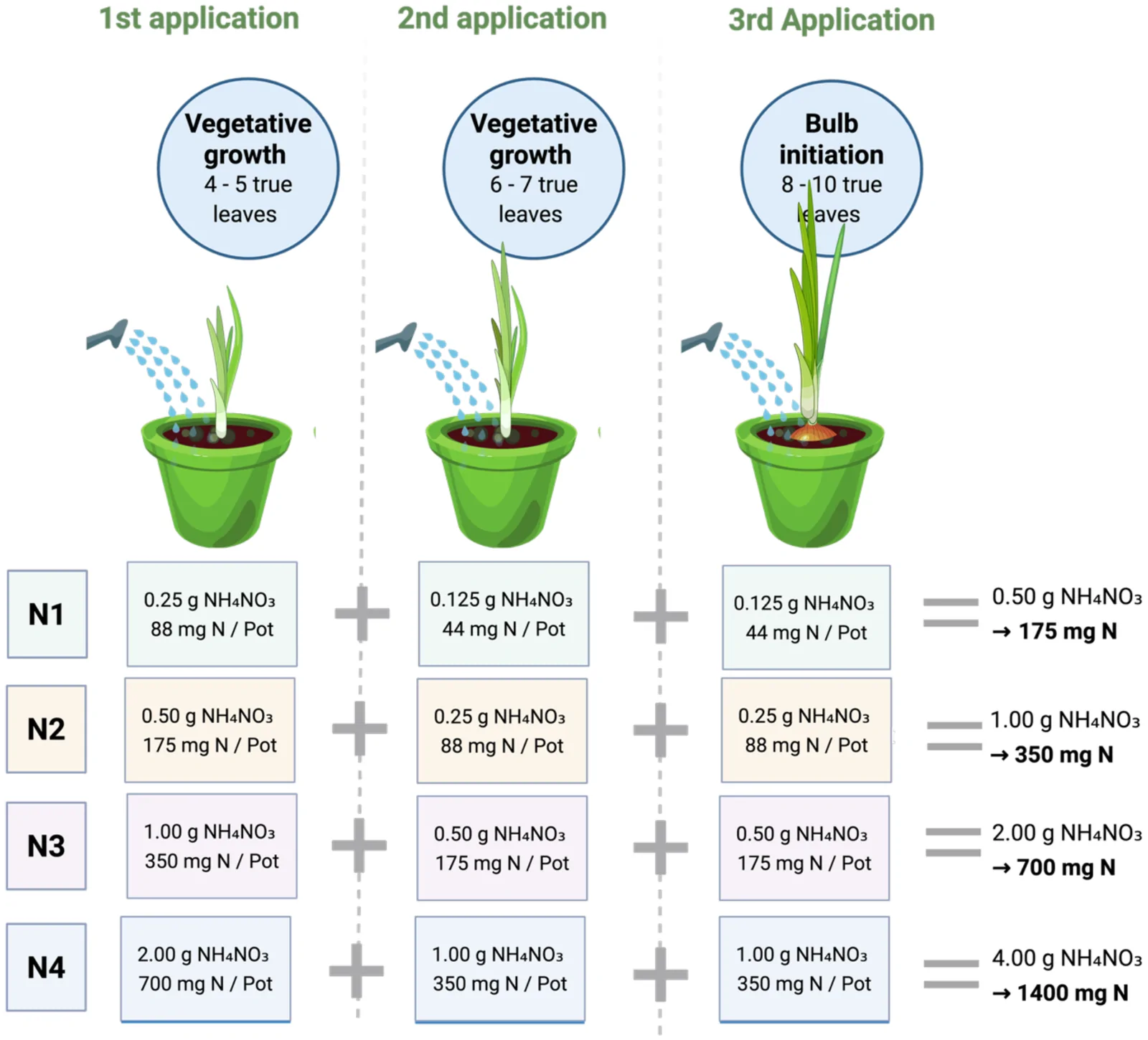
Nitrogen treatments (N1–N4) and timing of three split applications of NH_4_NO_3_ during the pot experiment. Amounts refer to total NH_4_NO_3_ applied per pot; n = 6 pots per N treatment.

Four nitrogen supply levels (N1–N4) were established using ammonium nitrate (NH_4_NO_3_) as the nitrogen source. The applied amounts refer to the total mass of NH_4_NO_3_ per pot, with the corresponding amount of elemental nitrogen given in parentheses. Thus, N1 received 0.50 g NH_4_NO_3_ pot^-^¹ (equivalent to 175 mg N pot^-^¹); N2 received 1.00 g NH_4_NO_3_ pot^-^¹ (equivalent to 350 mg N pot^-^¹); N3 received 2.00 g NH_4_NO_3_ pot^-^¹ (equivalent to 700 mg N pot^-^¹); and N4 received 4.00 g NH_4_NO_3_ pot^-^¹ (equivalent to 1400 mg N pot^-^¹). The fertilizer was supplied in three split applications (**Figure 1**). The split application design was chosen to approximate agronomic nitrogen management, maintain nitrogen availability across establishment, vegetative growth, and bulb initiation, and avoid an excessive initial salt or nitrogen pulse in the pot system. This approach was also aligned with observations from a preliminary experiment conducted in 2021. The first application was given immediately after transplanting to ensure sufficient nitrogen availability during plant establishment. The second application followed two to three weeks later to promote optimal vegetative growth, and the third application was performed shortly before bulb initiation to support bulbing and subsequent maturation.

At each application, NH_4_NO_3_ was dissolved in water and evenly distributed on the soil surface. Pots were irrigated regularly to maintain uniform soil moisture and minimize leaching. The experiment followed a completely randomized design with six biological replicates per nitrogen level and variety.

### 2.3 Sampling and biochemical analyses

During the growing period, the number of leaves per plant was recorded to monitor vegetative development. At maturity, plants were harvested and bulbs were air-cured for three weeks in a dry, well-ventilated room. After the curing period, bulbs were separated from the foliage and cleaned of residual soil and loose outer dry scales where necessary. For each pot, mean bulb weight, bulb height, and bulb diameter were recorded to assess morphological responses to the nitrogen treatments.

Soil samples were collected from each pot immediately after harvest, stored at 4 °C, and later analyzed for mineral nitrogen (N_min_), total nitrogen (N), sulfur (S), and carbon (C) concentrations.

Foliage was collected separately from the bulbs. Since the foliage was already partially desiccated after the curing period, it was collected in dry form and subsequently oven-dried at 65 °C for 42 h in a ventilated oven. Dried leaf material was finely ground using a ball mill (MM 301, Retsch, Haan, Germany; 60 s at 30 Hz) and used for the determination of total carbon (C), nitrogen (N), and sulfur (S) concentrations.

For bulb analyses, the four bulbs harvested from each pot were pooled and treated as one biological replicate. After morphological measurements, pooled bulb material from each pot was divided into representative subsamples according to the requirements of the subsequent analyses. Subsamples intended for metabolite, biochemical, and elemental analyses were immediately shock-frozen in liquid nitrogen, freeze-dried, and ground to a fine powder. This freeze-dried bulb powder was used for metabolomic profiling as well as for the determination of total phenolic content, antioxidant capacity, flavonol compounds, mineral nitrogen (N_min_), total nitrogen (N), sulfur (S), and carbon (C). Additional pooled bulb subsamples were processed separately for analyses performed on fresh material, including pyruvic acid, soluble solids, non-structural carbohydrates, and dry matter determination.

For the fresh-material bulb analyses, representative fresh bulb tissue from each pooled pot sample was divided into two subsamples. One subsample was homogenized 1:1 (w/v) with double-distilled water using a BÜCHI Mixer B-400 and filtered through filter paper (520 A ½, Schleicher & Schuell). The filtrate was stored at −20 °C until pyruvic acid determination. The second subsample was homogenized without dilution and used for the determination of soluble solids, non-structural carbohydrates, and dry matter.

For the wet-chemical bulb analyses, representative fresh bulb tissue from each pooled pot sample was divided into two subsamples. One subsample was homogenized 1:1 (w/v) with double-distilled water using a BÜCHI Mixer B-400 and filtered through filter paper (520 A ½, Schleicher & Schuell). The filtrate was stored at −20 °C until pyruvic acid determination. The second subsample was homogenized without dilution and used for the determination of soluble solids, non-structural carbohydrates, and dry matter.

Dry matter content was determined gravimetrically by oven-drying 20 g of homogenized bulb tissue at 70 °C for 42 h, followed by drying at 105 °C for 3 h (Romo-Perez et al., 2020). The initial lower-temperature step was used to remove most water gently from sugar-rich onion tissue, while the final 105 °C step was applied to remove residual moisture before calculating dry matter. Total soluble solids were measured at 22 °C using handheld and laboratory refractometers.

Non-structural carbohydrates, including reducing sugars, sucrose, and fructans, were extracted in hot water at 95 °C. Samples were homogenized using an Ultra-Turrax, diluted to 20 mL, and centrifuged at 10,000 × g for 15 min at 25 °C. The resulting supernatant was used for further analyses. Quantification was performed using a modified PAHBAH assay according to McCleary et al. (2000). Reducing sugars were measured directly from diluted extracts (1:5). Sucrose, as a non-reducing sugar, was enzymatically hydrolyzed using sucrase in sodium maleate buffer (pH 6.5, 40 °C, 30 min) prior to PAHBAH derivatization. Fructans were quantified after acid hydrolysis with 1 M HCl at 95 °C for 4 min, followed by dilution (1:2) and colorimetric determination. A glucose calibration curve ranging from 0 to 1 g L^-^¹ was used for quantification, and concentrations were expressed as mg g^-^¹ fresh weight (FW). Total sugars (TS) were calculated as the sum of reducing sugars, sucrose after enzymatic hydrolysis, and fructans after acid hydrolysis. This definition follows the analytical framework applied in previous studies on onion carbohydrate reserves (Romo-Perez et al., 2020, 2024).

Pyruvic acid, used as an indicator of onion pungency, was determined using the DNPH-based colorimetric assay described by Anthon and Barrett (2003), with onion sample preparation and background pyruvate correction based on Yoo and Pike (2001). Briefly, fresh bulb tissue was homogenized 1:1 (w/v) with double-distilled water, filtered, and stored at −20 °C until analysis. For quantification, 25 µL of filtrate were incubated with 1 mL double-distilled water and 1 mL DNPH solution (12.5 mg DNPH in 50 mL 1 M HCl) at 37 °C for 10 min. Subsequently, 1 mL of 1.5 M NaOH was added. Absorbance was measured at 515 nm, and concentrations were calculated using a sodium pyruvate standard curve ranging from 0 to 8 mM. Background pyruvate levels were determined and subtracted according to Yoo and Pike (2001) to obtain enzymatically developed pyruvate values.

Total phenolic content and antioxidant capacity were determined from freeze-dried onion bulb powder. For extraction, 75 mg of powder was mixed with 0.75 mL of 75% ethanol (ethanol:water, 75:25, v/v). Samples were magnetically stirred for 30 min at room temperature and subsequently treated in an ultrasonic bath for 20 min. After centrifugation at 3000 × g for 15 min, the supernatant was collected. The extraction was repeated twice, after 45 min and 90 min, and the combined extracts were stored at −80 °C in the dark until analysis to prevent light-induced oxidation.

Antioxidant capacity was determined using a DPPH radical scavenging assay. A 0.1 mM DPPH solution was prepared in 75% ethanol. Trolox standards ranging from 0 to 400 µM were prepared from a 2 mM stock solution. Aliquots of 0.1 mL of standards or diluted extracts were mixed with 1 mL of DPPH reagent and incubated for 1 h in the dark at room temperature. Absorbance was then measured at 515 nm. Results were expressed as µmol Trolox equivalents (TE) g^-^¹ dry weight (DW).

Total phenolic content (TPC) was quantified using the Folin –Ciocalteu method adapted for microplate assays. Briefly, 60 µL of ethanolic extract were mixed with 450 µL of 1:10 diluted Folin-Ciocalteu reagent and incubated for 10 min. Subsequently, 450 µL of 2% (w/v) Na_2_CO_3_ solution was added. Samples were kept in the dark at room temperature for 45 min. Absorbance was measured at 765 nm in triplicate using a microplate reader. Gallic acid standards ranging from 0 to 200 mg L^-^¹ were used to construct a calibration curve, and results were expressed as mg gallic acid equivalents per gram dry weight (mg GAE g^-^¹ DW).

Flavonol concentrations, including quercetin derivatives, were quantified in freeze-dried bulb tissue as described by Romo-Perez et al. (2024). Briefly, 100 mg of freeze-dried bulb powder was extracted with 80% ethanol, shaken for 3 h at room temperature, and centrifuged at 14,000 × g for 10 min. The supernatant was filtered through a PTFE filter and stored at −80 °C until analysis. Quercetin-4′-O-glucoside, quercetin-3,4′-O-diglucoside, isoquercetin, and free quercetin were determined by HPLC using a VWR/HITACHI Chromaster 5000 system equipped with a Zorbax Eclipse Plus C18 column (250 mm × 4.6 mm, 5 µm; Agilent). Detection was performed at 360 nm.

### 2.4 Elemental carbon, sulfur, nitrogen; soluble sugars and amino acid analyses

Total carbon (C), nitrogen (N), and sulfur (S) in leaf and bulb tissues were determined by dry combustion using an Elementar Vario EL analyzer (Elementar Analysensysteme GmbH, Langenselbold, Germany), following VDLUFA (2000). The elemental C:N ratio was calculated from total carbon and total nitrogen contents. Because total carbon includes both structural and non-structural organic carbon pools, the C:N ratio was interpreted as an integrative indicator of elemental tissue composition rather than as a direct measure of metabolically active carbon allocation.

Soil mineral nitrogen (N_min_ = NO_3_^-^ + NH_4_^+^) was quantified colorimetrically using a continuous flow analyzer (Alliance Instruments, Salzburg, Austria) according to VDLUFA (2018). Soil samples were extracted in 125 mM CaCl_2_, shaken, and filtered prior to analysis. Nitrate was reduced with hydrazine sulfate and copper, while ammonium was determined via indophenol dye formation. Absorbance was measured at 540 nm (NO_3_^-^) and 660 nm (NH_4_^+^). Citrate was added as a complexing agent to minimize interference from alkaline earth metals. Results were expressed as area-based mineral nitrogen content (N_min_ mg kg^-1^), adjusted for sampling depth, bulk density, and dry matter content. The same CaCl_2_ extracts were also analyzed for soluble sulfur (S_min_) using inductively coupled plasma optical emission spectrometry (ICP-OES, Agilent 5110, Santa Clara, USA).

Nitrate concentrations in plant tissues (bulb) were determined by ion chromatography (Dionex Integrion HPIC, Thermo Fisher Scientific) following aqueous extraction (50 mg freeze-dried material in 5 mL H_2_O). Separation was achieved using an IonPac AS11-HC column under hydroxide gradient elution and conductivity detection. Quantification was based on a certified multi-point calibration (Carl Roth GmbH) using Chromeleon 7 software. Nitrate concentrations were measured in freeze-dried bulb material and are reported on a dry-weight basis (mg NO_3_^-^ kg^-^¹ DW).

Free amino acids were analyzed using ion exchange chromatography with post-column derivatization (Biochrom 30+, Biochrom Ltd., Cambridge, UK). Detection was carried out at 570 nm (primary amino acids) and 440 nm (secondary amino acids). The procedure followed Commission Regulation (EC) No 152/2009 (European Commission, 2009) and included certified reference material (NIST SRM 2389a) for quality assurance.

Soluble sugars and sugar alcohols were determined by anion-exchange chromatography with pulsed amperometric detection (Dionex ICS-5000+, Thermo Fisher Scientific) using a CarboPac MA1 column, following Thermo Fisher Application Note 122 and the method described by Menge-Hartmann et al. (2009).

Analyses of total C, N, S, soil mineral nitrogen, soluble sulfur (S_min_), plant nitrate, free amino acids, and soluble sugars were conducted by the Core Facility of the University of Hohenheim.

### 2.5 Untargeted GC×GC–MS metabolomics and data processing

Untargeted GC×GC–MS-based metabolite profiling was essentially performed as in earlier works on onions (Romo-Pérez et al., 2018; Romo-Perez et al., 2021, 2024), but with some modifications. Briefly, twenty mg of freeze-dried and finely ground bulb material were extracted twice with 750 µL methanol, derivatized with 20 µL methoxylamine-hydrochloride in pyridine (20 mg mL^-^¹) and 50 µL MSTFA, and analyzed using an apolar × medium-polar column setup in a GC×GC-qMS system. The column setup consisted of an Rxi-5SilMS first-dimension column (30 m plus 7 m integrated pre-column × 0.25 mm i.d. × 0.25 µm film thickness; Restek, Bellefonte, PA, USA) coupled to a BPX50 second-dimension column (2.0 m total length, including a 0.7 m separation segment × 0.15 mm i.d. × 0.15 µm film thickness; Trajan Scientific, Ringwood, Victoria, Australia). Further details on sample extraction, derivatization, instrument configuration, temperature programs, modulation settings, and MS acquisition parameters are provided in the **Supplementary Material**, Section 1.

Raw data were extracted with Shimadzu GCMSsolution v. 4.45 and compiled as one peak list per chromatogram comprising all integrated peaks and the results of automated compound annotation using an in-house library and FAME-based retention indices. Further processing, including data aggregation, filtering, alignment, and QC sample-based drift and offset correction, was performed with R scripts developed in-house (Egert et al., 2015). Analyte variables selected based on statistical analyses were further evaluated, and compound identity was manually assessed in a two-step procedure. First, spectra were matched against an in-house library established by the injection of reference compounds into the same GC×GC–MS platform. In this database comparison, retention indices based on saturated fatty acid methyl esters were also taken into account, with the deviation between library RI and measured RI usually being <|5| units. When this was unsuccessful, matching was also performed against the NIST2023 and FiehnLib databases.

Special attention was paid to the identification of ACSOs. When subjected to the common methoximation/trimethylsilylation protocol, these compounds form multiple but reproducible derivatives. The in-house library contained spectra of alliin, deoxyalliin, isoalliin, and methiin. The spectra of these compounds usually exhibited ions characteristic of TMS derivatives of amino acids, such as m/z 218, often in combination with other ions apparently more specific for ACSO derivatives, e.g., m/z 160, 166, 186, or 188. Of the known ACSOs, isoalliin was most abundant in the onion samples, followed by methiin, while alliin and deoxyalliin were not detected. In addition, several other compounds were observed whose spectra were similar to those of known ACSOs and which showed similar responses to the experimental factors. These metabolites were therefore reported as putatively annotated “ACSO-like” compounds (MSI level 3). The ACSO-related variables from the untargeted GC×GC–MS dataset were evaluated on a relative-quantitative basis and should not be interpreted as absolute concentrations of individual cysteine sulfoxides.

### 2.6 Statistical analysis

#### 2.6.1 Morphological and quality parameters

Morphological and quality traits were analyzed using two-way ANOVA with variety, nitrogen treatment, and their interaction as fixed effects. Type-III ANOVA was used to evaluate the main effects and the variety × nitrogen interaction. Estimated marginal means were calculated from the full model. For graphical presentation, Tukey-adjusted comparisons among nitrogen treatments were performed within each variety and shown as compact letter displays in the figures. Formal interpretation was based on the two-way ANOVA results: variety-specific nitrogen responses were interpreted when the variety × nitrogen interaction was significant, whereas significant nitrogen effects without a significant interaction were interpreted as main treatment effects across varieties. All pairwise comparisons and planned contrasts were two-sided; ANOVA F-tests were evaluated using the standard upper-tail F test. Statistical significance was assessed at α = 0.05.

#### 2.6.2 Metabolites and elemental C:N status variables

For metabolites, including carbohydrates, amino acids, organic acids, and secondary metabolites, as well as N- and C-status parameters, two-way ANOVA models were fitted with variety, nitrogen level, and their interaction as fixed effects. The interaction term was included to test whether the response to nitrogen differed between the two varieties. Type-III ANOVA was used to evaluate each factor while accounting for the other terms in the model. The analyses were based on n = 6 pots per nitrogen treatment. Model assumptions were checked using Q–Q plots and residual-versus-fitted plots. When necessary to stabilize variance or reduce skewness, for example for some amino acids, data were log-transformed before analysis. Results were then back-transformed and reported on the original scale.

Model-estimated treatment means, also referred to as estimated marginal means (EMMs), were calculated for each nitrogen level within each variety. These means were used for Tukey-adjusted pairwise comparisons between nitrogen levels within each variety (α = 0.05).

Bivariate associations with the nitrogen gradient were evaluated using Pearson correlations on z-standardized variables. Confidence intervals were derived using Fisher’s z-transformation, and multiple testing was controlled using the Benjamini-Hochberg false discovery rate (FDR). Correlation networks were based on Pearson correlations between standardized variables and filtered by |r| ≥ 0.75 and FDR ≤ 0.01. Nodes represent metabolites or traits, and edge signs indicate positive or negative associations.

All analyses were conducted in R version 4.3.3. ANOVA was performed using the car package (Fox and Weisberg, 2019). Estimated marginal means and Tukey-adjusted comparisons were calculated using emmeans (Lenth and Piaskowski, 2025) with compact letter displays generated using multcomp (Hothorn et al., 2008). Graphics were produced using ggplot2 (Wickham, 2016).

#### 2.6.3 Principal component analysis

A principal component analysis (PCA) was performed on the quantitative trait matrix (bulb morphology, quality markers, targeted metabolites, and metabolites from the untargeted metabolomics dataset). Before PCA, variables were centered and scaled to unit variance. Missing values were checked before PCA; the single missing value detected in the PCA matrix was imputed using the median of the corresponding variable, so that no sample or variable was excluded due to missing values. PCA scores were visualized by variety and N treatment; loadings were shown separately as arrows, with arrow length proportional to the variable’s contribution to the first two components. PCA was computed with prcomp (R base stats); plots were created with ggplot2.

To quantify which variables are most influenced by experimental factors, we computed eta-squared (η²) effect sizes from fixed-effects ANOVA for each variable separately: treatment, treatment*variety and variety.

We used classical (non-partial) η² (partial = FALSE), ensuring that η² for treatment, variety, interaction, and the residual fraction sum to 1 per variable. Variables were ranked by η², and the mean η² across variables was summarized in a stacked bar to depict the relative contribution of treatment, variety, interaction, and unexplained variance. Effect sizes were obtained with effect size (Ben-Shachar et al., 2020); compact visualizations used ggplot2.

#### 2.6.4 Heatmaps

For each variety, heatmaps were generated from log_2_ fold changes (log_2_FC) of each variable relative to the N2 treatment (1 g NH_4_NO_3_). Where zero values occurred, a variable-specific pseudo-count equal to half the smallest positive observation of the respective variable was added before log transformation. This was done solely to enable log transformation of zero values. Using a variable-specific pseudo-count minimized distortion across metabolites with different absolute abundance ranges, because the correction was scaled to the smallest non-zero value within each variable. For each nitrogen level (N1, N3, N4), Welch’s two-sample t-test was performed against N2 within each variety on log_2_-transformed data. P-values were adjusted within each heatmap column using the Benjamini-Hochberg FDR. Heatmaps were produced using pheatmap (Kolde, 2019), with optional dendrogram adjustment via dendextend (Galili, 2015).

## 3 Results

### 3.1 Morphological and quality traits

**Figure 2:**
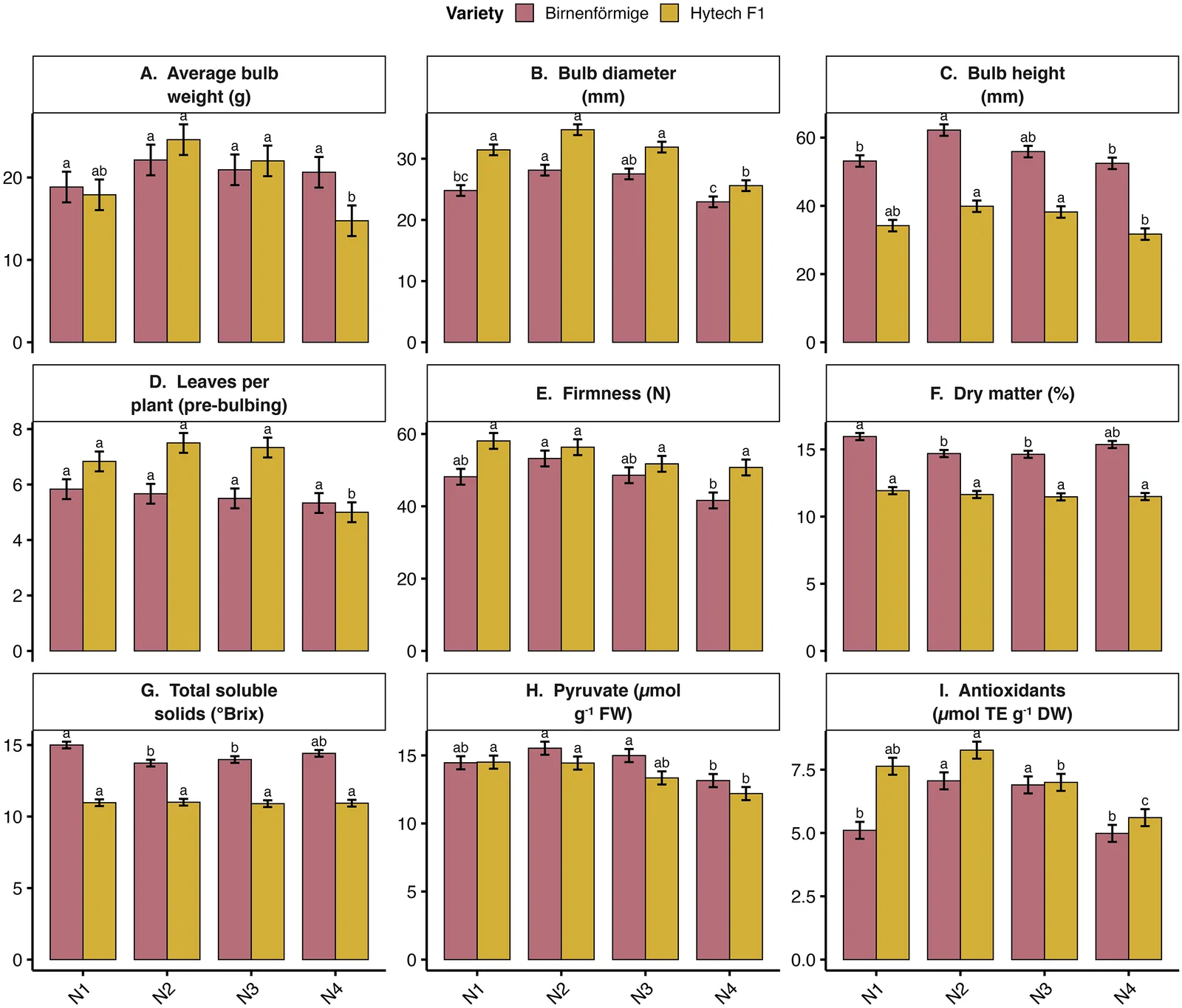
Morphological and quality parameters of variety Birnenförmige (pink) and variety Hytech F1 (gold) onions grown under four nitrogen supply levels (N1–N4: 0.5, 1.0, 2.0, and 4.0 g NH_4_NO_3_ per pot). **(A)** Average bulb weight per plant, calculated per pot (yield proxy), **(B)** bulb diameter, **(C)** bulb height, **(D)** leaves per plant recorded prior to bulb initiation, **(E)** bulb firmness, **(F)** dry matter content, **(G)** total soluble solids (°Brix), **(H)** pyruvate concentration (µmol g^-^¹ FW; pungency marker), and **(I)** antioxidant capacity (µmol TE g^-^¹ DW). Bars represent means ± SE (n = 6 biological replicates; each replicate = one pot with four pooled plants). Different letters indicate significant differences among nitrogen treatments within each variety based on Tukey-adjusted comparisons from the full two-way ANOVA model. Formal two-way ANOVA results are provided in **Supplementary Table 1**.

Bulb weight responded to nitrogen level (two-way ANOVA, p = 0.0126), while the variety main effect and the interaction were not significant (variety: p = 0.535; variety × treatment: p = 0.139). Full two-way ANOVA results for all morphological and quality traits are provided in **Supplementary Table 1**. Within Birnenförmige, estimated marginal means were 18.8–22.1 g across N1–N4 and did not differ by Tukey HSD. Within Hytech F1, bulb weight peaked at N2 (24.6 g) and declined sharply at N4 (14.8 g). The difference between N2 and N4 was significant (Tukey HSD, p < 0.05). Between varieties, a significant difference was observed only at N4, with Birnenförmige producing heavier bulbs than Hytech (p = 0.031; **Figure 2A**). Overall, bulb weight was highest at intermediate nitrogen supply and declined at N4, with this pattern being more pronounced in Hytech, whereas Birnenförmige remained comparatively stable across the N levels.

Bulb diameter (**Figure 2B**) was significantly affected by both variety and nitrogen level, while the interaction was not significant. Across nitrogen levels, Hytech consistently produced larger bulb diameters than Birnenförmige. Within both varieties, bulb diameter increased from N1 to N2–N3, followed by a slight reduction at N4, indicating a plateau or decline at high nitrogen supply. Bulb height (**Figure 2C**) showed significant main effects of variety (p < 0.001) and nitrogen level (p < 0.001), but no interaction (p = 0.54). Birnenförmige had consistently taller bulbs than Hytech across all nitrogen levels. For both varieties, bulb height was lowest under N1 and increased to N2-N3, suggesting that moderate nitrogen availability promoted vertical bulb growth, while no further increase occurred at the highest level.

Leaf number (**Figure 2D**) was significantly affected by variety, nitrogen level, and their interaction (variety, p < 0.001; treatment, p = 0.001; interaction, p = 0.013). Hytech generally produced more leaves than Birnenförmige, particularly under intermediate nitrogen levels (N2–N3; Δ = 1.83 leaves, SE = 0.51; p = 0.0008 for both). In contrast, Birnenförmige showed a consistently lower but more stable leaf number across treatments, whereas Hytech exhibited a decline at high (N4) nitrogen supply. This pattern explains the significant interaction, reflecting the greater nitrogen sensitivity of Hytech compared with the more conservative response of Birnenförmige.

Bulb firmness (**Figure 2E**) differed between varieties (p < 0.001) and varied with nitrogen level (p = 0.002), while the interaction was not significant. Across treatments, Hytech bulbs were generally firmer than those of Birnenförmige. In Birnenförmige, firmness slightly increased from low to intermediate nitrogen but declined at the highest level (N4), whereas Hytech showed the highest firmness at N1–N2 and a gradual, non-significant decrease with increasing nitrogen supply.

Dry matter (**Figure 2F**) was strongly variety-dependent, with Birnenförmige showing consistently higher values than Hytech across all nitrogen levels (p < 0.001). Dry matter was also affected by nitrogen level, while the interaction was not significant. Birnenförmige displayed slightly lower dry matter at intermediate levels (N2–N3). In contrast, Hytech exhibited a gradual decline in dry matter with increasing N supply, a pattern consistent with the shift from carbon-storage traits toward nitrogen-rich composition discussed in Section 4.2.

Quality-related traits are shown in **Figures 2G–I**. Total soluble solids differed strongly between varieties (Birnenförmige > Hytech; p < 0.001), whereas neither the nitrogen main effect nor the interaction reached significance (p = 0.053 and p = 0.051, respectively; **Figure 2G**). Pyruvate concentration is a marker of pungency and decreased significantly with increasing nitrogen (p < 0.001), with Birnenförmige maintaining higher values than Hytech (variety effect p = 0.011; **Figure 2H**). Antioxidant capacity decreased significantly with nitrogen (p < 0.001) and showed a significant nitrogen × variety interaction (p = 0.0056), indicating an earlier and stronger decline in Hytech, with values already reduced at N3, whereas Birnenförmige showed a more gradual decrease (**Figure 2I**).

Representative bulbs of both varieties illustrate consistent morphological differences and their responses to nitrogen supply (**Supplementary Figure 1**). The hybrid Hytech F1 produced compact, round bulbs that were largest at moderate N supply (N2) but decreased in size and weight under higher N levels (N3–N4). In contrast, the traditional variety Birnenförmige maintained its elongated bulb shape up to N3, with a visible reduction in bulb size at the highest N level (N4).

### 3.2 Variety effects dominate, while nitrogen causes shifts within each variety (PCA, η²)

**Figure 3:**
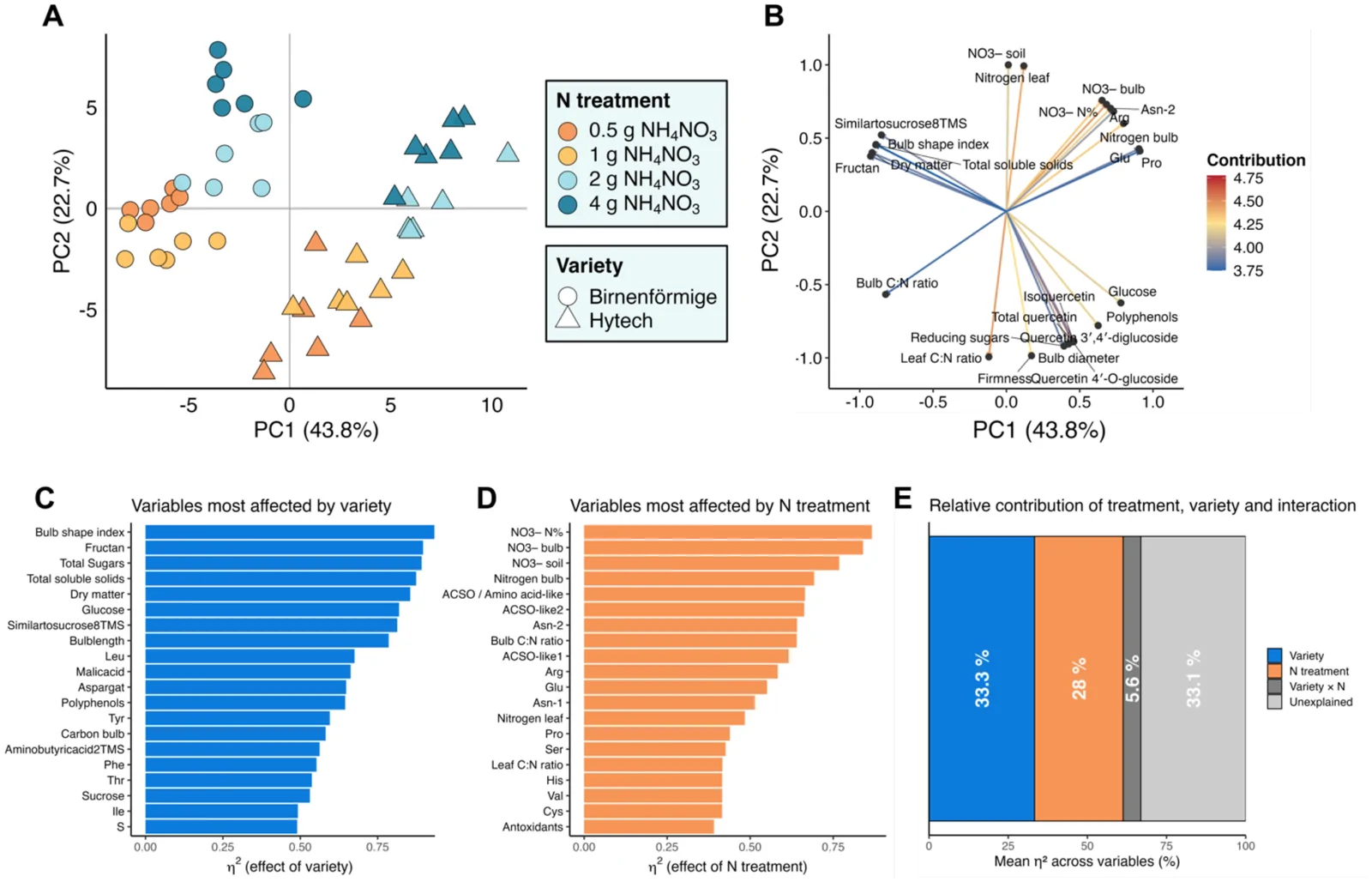
Principal component analysis (PCA) and variance partitioning (η²) of morphological and metabolic traits. **(A)** PCA scores showing clear separation between varieties (PC1) and ordered gradients across N treatments (PC2). **(B)** PCA loadings indicating main contributors to the two principal components. **(C, D)** Ranked η² values identifying variables most affected by **(C)** variety and **(D)** N treatment. **(E)** Mean proportion of total variance attributed to variety, N treatment, their interaction, and unexplained variance.

The PCA clearly separated the two varieties along PC1, while N levels formed ordered gradients within each variety along PC2 (**Figure 3A**). The loadings panel indicates that morphology- and quality-related traits (bulb size, total soluble solids, dry matter, sugars) loaded with the variety axis, whereas N-status indicators (nitrate pools, total N, several amino acids) aligned with the N gradient (**Figure 3B**).

Trait-wise η² values (effect sizes) confirmed this pattern. The strongest variety effects occurred in morphology/quality and carbon-related traits such as bulb shape index, fructans, total sugars, total soluble solids, dry matter, glucose, and bulb length, together with several aromatic (phenylalanine, tyrosine) and aliphatic amino acids (leucine, isoleucine, threonine, alanine) that differed markedly between varieties (**Figure 3C**). In contrast, N treatment most strongly affected nitrate concentrations in bulb and soil, total N in bulb and leaves, asparagine (both derivatives), elemental bulb C:N ratio, and N-related amino acids (arginine, glutamate, proline), with additional effects on bulb diameter and organic acids such as citrate (C-metabolism) and pyruvate, the latter being a surrogate marker for pungency rather than stemming from central metabolism (**Figure 3D**). The variety × N interaction explained comparatively little variation overall; the clearest interactive responses were observed for bulb density, leaf number, reducing sugars/sucrose, several soil- and amino-N-related variables, and bulb weight (**Supplementary Figure 2**). On average across all variables, variety accounted for 33.3% of total variance, N treatment for 28.0%, and their interaction for 5.6%, leaving 33.1% unexplained (**Figure 3E**). Together, these results indicate a stable, variety-specific carbon/morphology baseline onto which a shared N response across varieties is superimposed, primarily shifting nitrate/N pools and N-rich amino acids.

### 3.3 Nitrogen status, carbon balance, and bulb metabolite profiles

**Figure 4:**
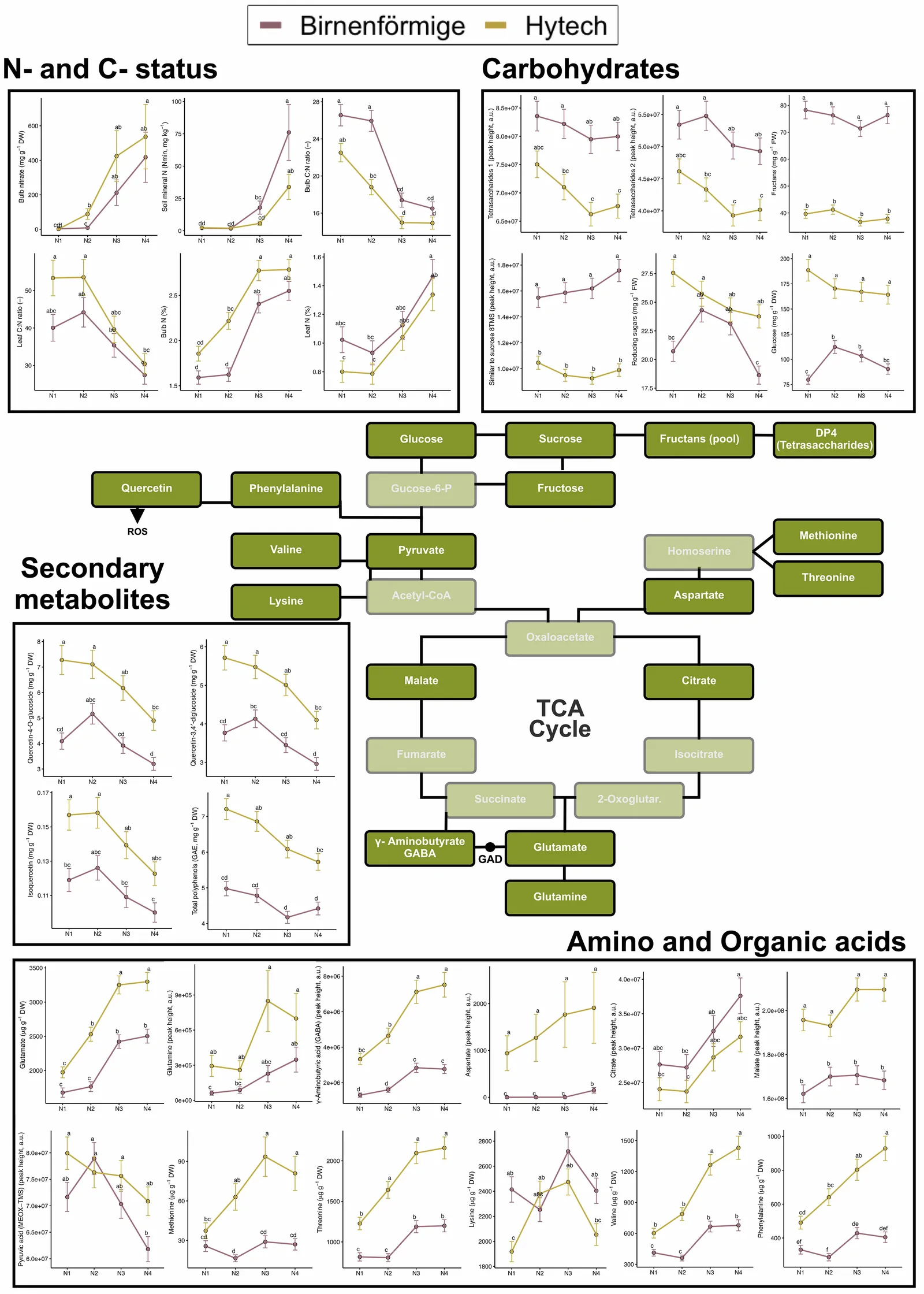
Nitrogen- and elemental carbon-related status traits and metabolite levels in bulbs of two onion varieties grown under increasing nitrogen supply (N1–N4). Data are grouped into four categories: N and elemental C status, carbohydrates, secondary metabolites, and amino/organic acids. Nitrate concentrations in bulb tissues are reported on a dry-weight basis (mg NO_3_^-^ kg^-^¹ DW). The central pathway map illustrates key metabolites involved in carbon and nitrogen metabolism. Significantly responsive metabolites are highlighted in green, while metabolites without significant changes are shown in light green. Values represent means ± SE.

**Figure 4** summarizes nitrogen- and carbon-related status traits together with bulb metabolite profiles across carbohydrates, amino acids and organic acids, and secondary metabolites for both varieties along the nitrogen gradient (N1–N4).

#### 3.3.1 N- and C-status

Nitrogen-related parameters showed strong treatment effects and clear variety differences (**Figure 4**; N- and C-status). Bulb nitrate concentrations were low under N1 in both varieties (≈ 2–3 mg kg^-^¹ DW) but increased strongly with rising nitrogen supply, reaching 537.4 mg kg^-^¹ DW in variety Hytech F1 and 417.7 mg kg^-^¹ DW in variety Birnenförmige under N4. Based on the corresponding dry matter contents, these values correspond to substantially lower fresh-weight concentrations. The onset of nitrate accumulation was earlier in variety Hytech F1, whereas variety Birnenförmige remained near-baseline at N1–N2 and increased more clearly from N3.

Residual soil mineral nitrogen (N_min_) at harvest highlighted contrasting uptake and utilization patterns (**Figure 4**; N- and C-status). Under N1 and N2, N_min_ remained similarly low in both varieties (≈ 2 mg kg^-^¹). With increasing nitrogen supply, variety Hytech F1 maintained lower residual N_min_, reaching 33.9 mg kg^-^¹ at N4, while variety Birnenförmige accumulated markedly more residual mineral nitrogen (76.1 mg kg^-^¹ at N4), consistent with lower uptake and/or lower sink demand under surplus conditions.

Elemental carbon–nitrogen ratios in bulb and leaf tissues further reflected distinct compositional patterns (**Figure 4**; N- and C-status). Bulb C:N ratio decreased with nitrogen in both varieties (from 26.6 to 16.5 in variety Birnenförmige; from 22.5 to 14.9 in variety Hytech F1), while remaining consistently higher in variety Birnenförmige across the gradient. In leaves, C:N ratio declined with increasing nitrogen in both varieties and converged at N4. Leaf nitrogen increased with nitrogen supply in both varieties and was consistently higher in variety Birnenförmige, whereas bulb nitrogen rose in both but reached higher values in variety Hytech F1 under high nitrogen. Together, these traits indicate contrasting N partitioning and elemental tissue composition, with relatively higher foliar N in variety Birnenförmige and stronger bulb N enrichment in variety Hytech F1 under surplus nitrogen.

#### 3.3.2 Carbohydrates

Carbohydrate profiles differed strongly between varieties and showed distinct response timing along the nitrogen gradient (**Figure 4**; Carbohydrates). Reserve-type pools (fructan pool and DP4/tetrasaccharides) were consistently higher in variety Birnenförmige than in variety Hytech F1. In variety Birnenförmige, these pools were relatively stable from N1 to N2 and declined more clearly from N3 onward. In contrast, variety Hytech F1 showed an earlier and more pronounced decline, with reductions already apparent between N1 and N2 and persistently low levels thereafter.

Soluble sugars (**Figure 4**; Carbohydrates) followed a different pattern. Variety Hytech F1 maintained higher reducing sugars and glucose across all N levels and showed a gradual decline with increasing nitrogen. Variety Birnenförmige showed increasing reducing sugars and glucose up to N2–N3, followed by a marked drop at N4. Overall, the carbohydrate data indicate that variety Birnenförmige maintained higher reserve-type pools and displayed delayed shifts under surplus nitrogen, whereas variety Hytech F1 showed earlier depletion of reserve pools and stronger reliance on soluble sugar dynamics (**Figure 4**; Carbohydrates).

#### 3.3.3 Amino and organic acids

Amino acids showed pronounced nitrogen responses, with consistently stronger and earlier increases in variety Hytech F1 (**Figure 4**; Amino and Organic acids). Glutamate and glutamine increased with nitrogen in both varieties, but reached higher levels in Hytech, especially at intermediate-to-high N. Several additional amino acids (including threonine, valine, methionine, and phenylalanine) were generally higher in Hytech and increased with nitrogen, whereas Birnenförmige showed more moderate or delayed increases (mainly from N3–N4). Lysine showed a compound-specific non-linear pattern in both varieties: Birnenförmige decreased from N1 to N2, peaked at N3, and declined at N4, while Hytech increased from N1 to N3 and dropped at N4.

Organic acids also differed between varieties (**Figure 4**; Amino and Organic acids). Malate was consistently higher in variety Hytech F1, while citrate showed stronger accumulation in variety Birnenförmige under high nitrogen. Aspartate exhibited strongly divergent dynamics, with high baseline levels and further increases in variety Hytech F1, but a delayed increase in variety Birnenförmige that became most apparent under N4.

#### 3.3.4 Secondary metabolites

Secondary metabolite profiles showed clear variety differences and distinct nitrogen response timing (**Figure 4**; Secondary metabolites). Variety Hytech F1 exhibited consistently higher flavonoid levels (quercetin derivatives and isoquercetin) than variety Birnenförmige. With increasing nitrogen, variety Hytech F1 showed a gradual decline across N1–N4. In contrast, variety Birnenförmige displayed a transient rise between N1 and N2 followed by a sharp decline from N3 onward. Total phenolic content mirrored these dynamics, with a steady downward trend in variety Hytech F1 and a more non-linear pattern in variety Birnenförmige.

### 3.4 Integrated metabolite-level responses along the nitrogen gradient and under N imbalance

**Figure 5:**
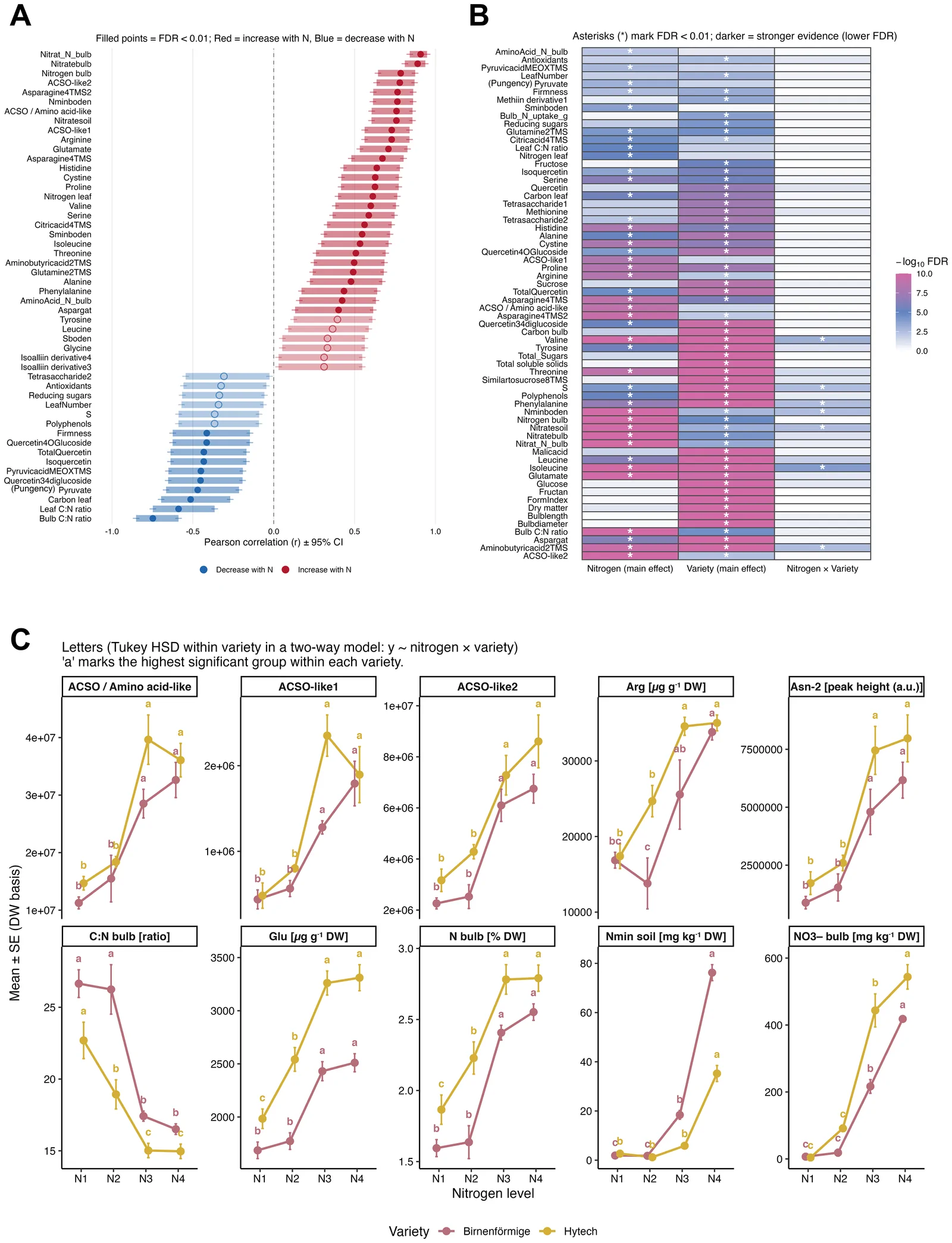
Metabolite-level responses along the nitrogen gradient in two onion varieties. **(A)** Pearson correlation coefficients (r) between nitrogen level (N1–N4) and standardized metabolite values across all samples. Filled points denote significant correlations (FDR < 0.01); red = increase with N, blue = decrease. **(B)** ANOVA per metabolite for the main effects of nitrogen and variety, and their interaction (nitrogen × variety). Asterisks indicate FDR < 0.01. **(C)** Nitrogen response curves (mean ± SE) for the ten most responsive metabolites, with Tukey groups (a–c) based on two-way ANOVA (y ~ nitrogen × variety). Letters are shown within each variety. ACSO-like compounds and isoalliin were relatively quantified by GC×GC–MS.

**Figure 6:**
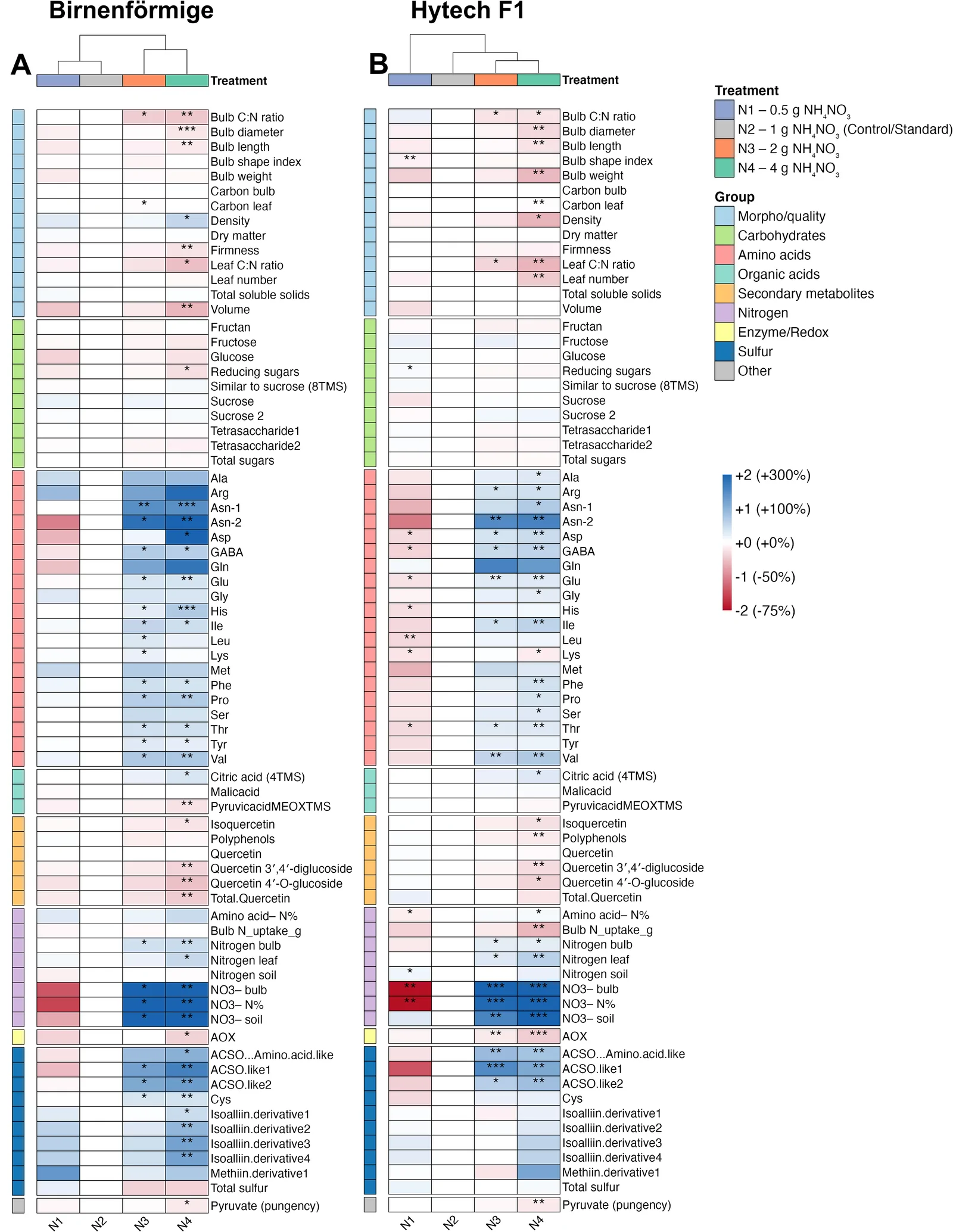
Log₂ fold changes (log₂FC) of selected metabolites and traits in two onion varieties, **(A)** Birnenförmige and **(B)** Hytech F1, under low nitrogen supply (N1), moderate nitrogen oversupply (N3), and high nitrogen oversupply (N4), each relative to the sufficient nitrogen level N2. Bars indicate mean log₂FC per treatment and variety; asterisks denote significance levels based on FDR-adjusted p-values (*q < 0.05, **q < 0.01, ***q < 0.001). Negative values indicate decreases under N imbalance, while positive values reflect accumulation or increases. Metabolites and traits are grouped by functional categories: nitrogen status, amino acids, and quality-related traits.

To identify coordinated response patterns across metabolites, correlation analyses, variance partitioning, and targeted contrasts were applied to standardized metabolite values (**Figures 5**, **6**).

#### 3.4.1 Coordinated shifts along the nitrogen gradient

Across all samples, **Figure 5A** shows strong positive associations between nitrogen level and nitrogen-rich soluble pools, including nitrate, glutamine, glutamate, asparagine, and multiple amino acids. In contrast, several carbon-associated and quality-linked traits were negatively associated with increasing nitrogen, including total phenolic content and quercetin derivatives, pyruvate (pungency marker), total sulfur in the bulb, and elemental leaf and bulb C:N ratios. Overall, the correlation pattern indicates a coordinated shift toward a more nitrogen-rich composition with increasing nitrogen supply.

Variance partitioning (**Figure 5B**) showed that variety explained more variation than nitrogen for many metabolites, particularly among amino acids (*e.g*., GABA, valine, isoleucine, phenylalanine) and secondary metabolites (quercetin derivatives, total phenolic content). Nitrogen explained substantial variation in core nitrogen-status traits (*e.g*., nitrate, asparagine, glutamate), while nitrogen × variety interactions were comparatively limited and concentrated in a subset of traits including GABA, valine, isoleucine, phenylalanine, total bulb sulfur, and soil N_min_.

**Figure 5C** illustrates representative response patterns across the nitrogen gradient using mean curves and Tukey groupings for the ten most responsive metabolites. Nitrogen-associated pools, including asparagine, annotated ACSOs and putatively annotated ACSO-like compounds, glutamate, and nitrate, increased more steeply with nitrogen in variety Hytech F1, whereas increases were more moderate and/or delayed in variety Birnenförmige. Conversely, elemental bulb C:N ratio decreased with nitrogen in both varieties but remained consistently higher in variety Birnenförmige (**Figure 5C**), indicating different baseline levels and response timing along the gradient.

To explore metabolite co-variation within each variety, correlation networks were constructed from pairwise Pearson correlations across nitrogen treatments (|r| ≥ 0.75, p ≤ 0.01) and clustered using Louvain community detection (**Supplementary Figure 3**). Nodes represent metabolites and edges represent significant correlations; modules therefore indicate sets of metabolites that co-vary tightly across the N gradient. In both varieties, amino acids and nitrogen-status traits (e.g., bulb N, nitrate in bulbs and soil, elemental bulb C:N ratio) formed densely connected modules, whereas carbohydrates, C-status traits, and flavonol glycosides tended to cluster separately. Network modularity (Q) was low overall but higher in variety Birnenförmige than in variety Hytech F1 (Q = 0.27 vs. 0.11), consistent with a clearer separation of metabolite clusters in variety Birnenförmige.

#### 3.4.2 Metabolite shifts under N deficiency and oversupply

To quantify responses under suboptimal nitrogen supply, log_2_ fold changes (log_2_FC) were calculated for N1, N3, and N4 relative to the sufficient level N2 (**Figure 6**). Under nitrogen deficiency (N1 vs. N2), variety Hytech F1 showed strong depletion of mobile nitrogen pools, with pronounced declines in bulb nitrate (log_2_FC −5.27, q < 0.01), NO_3_– N% (−5.07, q < 0.01), and amino acid–N% (−0.15, q < 0.05), alongside decreases in multiple free amino acids. In variety Birnenförmige, nitrate and amino acids shifted in the same direction but with smaller magnitude and fewer significant changes after FDR correction.

Under moderate oversupply (N3 vs. N2), variety Hytech F1 already accumulated nitrogen-rich metabolites (including glutamate, valine, isoleucine, asparagine, and nitrate), while most quality-related traits remained comparatively stable. In both varieties, several putatively annotated ACSO-like compounds began to increase at this stage, indicating early shifts in sulfur-associated metabolism under rising nitrogen supply. Variety Birnenförmige showed significant increases in several amino acids at N3, while bulb morphology remained largely unchanged.

Under high oversupply (N4 vs. N2), both varieties showed strong luxury accumulation patterns but with distinct emphasis. Hytech F1 accumulated nitrate strongly in bulbs and in NO_3_– N%, and many amino acids increased (including asparagine, glutamate, GABA, threonine, and valine). At the same time, multiple structural traits declined and pyruvate decreased, whereas putatively annotated ACSO-like compounds and isoalliin increased. Variety Birnenförmige followed the same overall directions but showed stronger increases in several sulfur-containing features and notable shifts in amino acids (including asparagine-2: 2.32, q < 0.01; aspartate: 6.04, q < 0.05), together with reductions in elemental bulb C:N ratio and quercetin derivatives under oversupply (**Figure 6**). Across both varieties, nitrate, asparagine, putatively annotated sulfur-related ACSO-like compounds, and selected amino acids (including GABA, valine, and phenylalanine) emerged as robust metabolite-level markers of nitrogen imbalance.

## 4 Discussion

### 4.1 Variety-specific baseline strategies – the physiological starting point

Across the full trait matrix, variety, rather than nitrogen supply, emerged as the dominant structuring factor. This is reflected in the PCA, where samples separated primarily by variety along PC1, whereas nitrogen levels formed an ordered gradient within each variety along PC2 (**Figures 3A, B**). Trait-wise η² values support the same pattern: on average, variety 33.3%, N treatment 28.0%, interaction 5.6%, unexplained 33.1% (**Figures 3C–E**).

At the trait level, landrace variety Birnenförmige consistently showed higher dry matter (**Figure 2F**), together with higher pools of fructans/oligosaccharides and a higher elemental bulb C:N ratio (**Figure 4**), supporting a pronounced carbon-storage signature at baseline. In contrast, hybrid variety Hytech F1 exhibited comparatively higher reducing sugars and glucose, as well as elevated levels of amino acids and several other nitrogen-status indicators (**Figure 4**), which is consistent with a more nitrogen-responsive metabolic baseline.

These contrasting baselines align with established onion carbohydrate biology. Fructans represent a major reserve carbohydrate in onion bulbs, and across cultivars, higher bulb dry weight is often associated with higher fructan content. At the same time, cultivar differences are well documented, with some varieties characterized by relatively low fructan pools but higher reducing sugars, underscoring that carbohydrate composition can vary substantially between genotypes (Darbyshire and Henry, 1979; Kleman et al., 2024). Importantly, the baseline pattern observed here, with higher fructan-related pools in variety Birnenförmige and higher reducing sugars in variety Hytech F1, has also been reported in earlier work (Romo-Perez et al., 2024; Romo-Pérez et al., 2026). Taken together, the two varieties start from different metabolic and elemental carbon–nitrogen “set points”, which helps explain why they reach critical thresholds under high nitrogen at different points.

### 4.2 Nitrogen supply reprograms primary metabolism via luxury uptake and carbon trade-offs

In both varieties, bulb biomass showed a non-linear response to nitrogen supply: biomass increased at intermediate levels (N2–N3) but declined at the highest level (N4). This decline was descriptively most pronounced in variety Hytech F1. Estimated bulb fresh weight peaked at 24.6 g at N2 and fell to 14.8 g at N4 (Δ = −9.8 g, −39.8%). In variety Birnenförmige, bulb weight changed only slightly (N2: 22.1 g vs N4: 20.6 g; Δ = −1.5 g, −6.8%) (**Figures 2A–C**). Thus, the pairwise difference at N4, where Birnenförmige outweighed Hytech F1, reflects the stronger high-N penalty in Hytech F1 rather than a generally higher yield potential of the landrace, and supports the interpretation of a more buffered biomass response in Birnenförmige under surplus N. Absolute bulb weights were small compared with field-grown commercial onion bulbs (**Supplementary Figure 1**), reflecting the pot-based experimental setup, limited rooting volume, and controlled treatment conditions; therefore, the biomass data should be interpreted primarily in relative terms across treatments and varieties. Such a decline at excessive N supply is consistent with previous controlled onion studies, where high nitrogen reduced bulb fresh weight and firmness (Randle, 2000), as well as with broader evidence that onion yield and quality responses can show diminishing returns at high N inputs (Geisseler et al., 2022).

A physiological explanation is that high nitrogen supply increases the demand for carbon and energy to assimilate nitrate and to build amino acids. This may reduce carbon availability for storage pools and alter the balance between carbon-rich reserves and soluble nitrogen forms, as reflected more directly by the fructan and soluble sugar data (Lawlor, 2002; Masclaux-Daubresse et al., 2010). In line with nitrogen supply exceeding plant demand at the highest level, soil mineral nitrogen (N_min_) increased strongly with nitrogen supply at harvest (**Figure 4**, N- and C-status), indicating substantial residual mineral nitrogen in the substrate. Overall, these patterns suggest that N4 did not just strengthen the response seen at N2–N3 but moved the plants into a condition where extra nitrogen and its metabolic cost no longer resulted in additional bulb biomass increase.

Bulb nitrate accumulation further supports the interpretation that N4 represented an oversupply condition with quality relevance. Nitrate concentrations measured in freeze-dried bulb material reached 537.4 mg kg^-^¹ DW in Hytech F1 and 417.7 mg kg^-^¹ DW in Birnenförmige under N4. Because these values are reported on a dry-weight basis, they should not be directly compared with fresh-weight regulatory or literature values. Considered on a fresh-mass basis, onion bulbs remain comparatively low nitrate accumulators relative to high nitrate-accumulating leafy vegetables, consistent with Santamaria’s classification of onion as a very low nitrate vegetable and with field-based data for storage onion (Santamaria, 2006; Golubkina et al., 2022). Current EU contaminant legislation sets nitrate maximum levels for selected high nitrate-accumulating vegetables such as spinach, lettuce, and rucola, as well as for baby foods, but not specifically for fresh onion bulbs (European Commission, 2023). Thus, the present nitrate data are best interpreted as a quality-relevant oversupply signal under pot conditions rather than as a regulatory compliance assessment. These findings support the recommendation that excessive nitrogen supply should be avoided when optimizing onion bulb yield and quality.

Organic acids and GABA provide an additional metabolite-level signature consistent with this oversupply phenotype. Citrate accumulated most clearly under high nitrogen in variety Birnenförmige, whereas malate remained consistently higher in variety Hytech F1 (**Figure 4**, amino acids and organic acids), suggesting genotype-specific routing of carbon skeletons within central metabolism. In parallel, GABA increased with nitrogen in both varieties and reached higher levels in variety Hytech F1 (**Figures 4**–**6**). Because GABA is closely linked to glutamate metabolism and often rises when N-rich soluble pools build up, this pattern is consistent with an “overflow/stress-associated” component under high N load, while remaining compatible with multiple underlying drivers. Unlike glutamate, glutamine, asparagine, and GABA, the non-linear lysine response is likely amino-acid-specific rather than a direct nitrate-assimilation signal and may reflect feedback-regulated amino acid metabolism or protein turnover; this pattern should therefore be interpreted cautiously.

This broader pattern of differential nitrogen responsiveness is consistent with observations in other crops, where landraces show smaller yield gains from high nitrogen than modern hybrids, indicating lower nitrogen responsiveness (and potentially greater yield stability across N levels) compared with hybrids (Ferro et al., 2006). Hytech F1 showed a stronger luxury-type accumulation in soluble N pools, with earlier and steeper increases in nitrogen-rich metabolites (notably amino acids) already at N2–N3 (**Figures 4**, **5C**, **6**), followed by a sharp decline in bulb biomass at N4 (**Figures 2A–C**). By contrast, Birnenförmige behaved more conservatively: changes in soluble N pools were delayed (mainly from N3 onward) and bulb biomass remained comparatively buffered across the N levels (**Figures 2**, **4**–**6**), consistent with a stronger fructan-rich storage background and a tighter coupling between nitrogen input and growth response.

Finally, under pot conditions, very high fertilizer rates may increase ionic strength of the substrate solution through salt accumulation. Because substrate electrical conductivity was not measured, this remains a speculative but plausible contributing factor to the N4 growth penalty (Robles-Aguilar et al., 2022; Méndez-Cifuentes et al., 2023).

### 4.3 Quality trade-offs under nitrogen imbalance: antioxidants and sulfur–pungency decoupling

Onion bulb quality is strongly shaped by composition. Key contributors include flavonoids (especially quercetin derivatives), sulfur-containing flavor precursors (ACSOs), and non-structural carbohydrates, and these groups respond differently to nitrogen supply (Griffiths et al., 2002).

The higher baseline levels of quercetin derivatives in Hytech F1 suggest that the two varieties differed in their inherent flavonol phenotype, rather than reflecting a simple hybrid-versus-landrace contrast. One possible explanation is the difference in bulb morphology: Hytech F1 formed more compact, round bulbs, whereas Birnenförmige produced more elongated bulbs, which may have influenced light exposure of outer bulb tissues and thereby flavonol formation. Additional factors, such as cultivar-specific outer-scale characteristics or differences in phenylalanine availability, may also have contributed. Because these factors were not tested separately, the higher flavonol levels in Hytech F1 should be interpreted as a variety-specific baseline difference, while the decline with increasing nitrogen represents an additional treatment effect.

**Figure 5A** shows that antioxidant-related traits were among the most consistent negative correlates of increasing nitrogen, including total phenolic content and quercetin derivatives. This pattern is reinforced by the log_2_FC analysis in **Figure 6**, where N oversupply (N4 vs. N2) was associated with marked decreases in flavonol glycosides and other phenolic antioxidant (**Figures 4**–**6**). At the same time, elemental bulb C:N ratios declined with nitrogen supply, while nitrogen increased in the soil (**Figure 4**), and the drop in flavonoid/phenolic traits occurred in parallel with this shift toward a more N-rich elemental composition. A similar link between nitrogen-driven decreases in elemental internal C:N balance and lower flavonoid levels has been reported under controlled N gradients, for example in *Cyclocarya paliurus* and *Coreopsis tinctoria* (Deng et al., 2019; Li et al., 2021). More broadly, work across horticultural crops supports that nitrogen management can shift antioxidant-related quality traits and that “optimal” quality often occurs at intermediate inputs rather than at maximal N supply (Stefanelli et al., 2010).

Notably, phenylalanine increased under N oversupply in our dataset (**Figure 6**), while flavonol glycosides and total phenolics decreased (**Figures 4**–**6**). This indicates that precursor availability did not limit flavonoid output; instead, pathway flux was likely constrained downstream of phenylalanine. However, metabolite pool sizes alone cannot resolve pathway flux, so this pattern points to regulatory control downstream of phenylalanine rather than precursor limitation. By analogy with transcriptional regulation described in *Arabidopsis* and apple, nitrate status may suppress parts of phenylpropanoid metabolism and affect flavonoid-related regulatory modules (Scheible et al., 2004; Wang et al., 2018). These studies provide a plausible mechanistic framework for the “Phe increases but flavonoids decrease” pattern observed here. However, direct evidence for nitrate-controlled MYB regulation or comparable transcriptional control of flavonoid biosynthesis in *Allium cepa* remains to be demonstrated. Together, these observations support interpreting the “Phe increases but flavonoids decrease” pattern as a putative regulatory decoupling under high N status, consistent with the metabolite-level trends shown in **Figures 5** and **6**. Finally, field studies in onion indicate that nitrogen fertilizer level does not always change quercetin content, highlighting that environment and genotype can buffer nitrogen effects (Mogren et al., 2006).

In parallel to phenylalanine, other soluble amino acids increased with nitrogen supply, most clearly the central N-assimilation hub (glutamine/glutamate) and the N-rich amide asparagine (**Figures 4**, **5A, C**, **6**), which is consistent with surplus nitrogen being buffered in free amino acid pools under high N supply (Masclaux-Daubresse et al., 2010; Deng et al., 2019). This build-up started earlier and more steeply in Hytech (already at N2), whereas Birnenförmige showed clearer increases mainly from N3 onward, reflecting variety-specific baseline metabolism and nitrogen responsiveness (**Figures 4**–**6**).

Carbohydrates still matter for bulb quality even when nitrogen effects are moderate. A large share of bulb dry matter consists of non-structural carbohydrates such as fructans and soluble sugars, which contribute to sweetness, texture, processing traits, and storage behavior (Griffiths et al., 2002). In the present dataset, carbohydrate profiles were mainly variety-driven, as **Figures 3C** shows: Birnenförmige maintained higher reserve-type pools (fructans pool and DP4/tetrasaccharides) together with higher dry matter, whereas Hytech showed higher soluble sugars (**Figure 4**, carbohydrates). Along the nitrogen gradient, carbohydrate shifts were present but generally less dominant than the responses of phenolics and sulfur-containing metabolites (**Figure 6**). Even so, the carbohydrate context links to the antioxidant pattern through elemental carbon–nitrogen status and non-structural carbohydrate pools: under graded nitrogen supply, rising nitrogen status, lower elemental C:N ratios, and lower flavonoid levels can occur in parallel (Stefanelli et al., 2010; Deng et al., 2019; Li et al., 2021).

Pyruvate, as a pungency proxy, showed an overall negative association with increasing nitrogen (**Figure 5A**), with the clearest decline occurring under the highest N level (**Figure 2H**), whereas isoalliin, known as the quantitatively most abundant ACSO in onions, as well as several putatively annotated ACSO-like compounds increased under oversupply (**Figures 5C**, **6**). The structures of these ACSO-like compounds cannot be easily resolved by GC–MS alone, because the characteristic sulfoxide group in the side chain can undergo degradation reactions during trimethylsilylation, resulting in multiple derivatives of the same compound. In addition*, Allium* sulfur metabolism includes γ-glutamyl peptide intermediates linked to ACSO/flavor precursor biosynthesis (Hughes et al., 2005). Because peptide bonds may also be cleaved during derivatization at elevated temperatures, some ACSO-like compounds responding to the nitrogen treatment may derive from peptide-related structures rather than from monomeric ACSOs. Analytical platforms that enable the detection of intact ACSOs, such as LC–MS, are therefore more suitable for targeted studies of ACSO metabolism.

All compounds measured by GC×GC–MS, including these ACSO-related compounds, were relatively quantified, while pyruvate reflects an enzymatically developed signal after tissue disruption. This divergence suggests, as a hypothesis-generating interpretation, that higher nitrogen may alter the profiles of sulfur-containing compounds without increasing the pyruvate-based pungency signal, consistent with a possible shift in ACSO-related composition and/or conversion efficiency rather than a simple “more sulfur = more pungency” relationship. Because enzymatically developed pyruvate integrates both precursor pools and post-disruption enzymatic conversion, changes in the relative abundance of different cysteine sulfoxides could potentially decouple ACSO-like compounds from pyruvate formation. Randle (2000) similarly showed that increasing nitrogen supply in hydroponically grown onions affected pungency-related quality and was associated with shifts in individual cysteine sulfoxides, indicating that onion flavor responses can reflect changes in ACSO composition rather than total sulfur accumulation alone.

The concomitant decline in total bulb sulfur in the present dataset (**Figure 6**) indicates that N oversupply altered internal N:S balance, consistent with hydroponic N×S work showing that nitrogen and sulfur availability interact to shape onion precursor profiles (Coolong and Randle, 2003). Field studies typically test agronomic N ranges and often report increasing pyruvate with N (Golubkina et al., 2022; Yeshiwas et al., 2024). In contrast, the present pot experiment included an N oversupply level beyond the productive range, which likely explains the downturn in pyruvate at N4.

### 4.4 Study boundaries and mechanistic outlook

Although onion is a globally important vegetable crop, relatively few studies have taken an integrative approach that captures nitrogen-driven responses across the major quality-related metabolic groups, including non-structural carbohydrates, phenolic antioxidants, and sulfur-containing compounds. This study leverages a controlled pot nitrogen gradient and a broad trait–metabolite matrix to resolve variety-specific physiological thresholds and coordinated quality trade-offs that are difficult to capture in field settings. In particular, the design spans an oversupply regime and links growth responses directly to shifts in nitrogen-rich soluble pools, antioxidant phenolics, and sulfur-related flavor-associated features, providing a mechanistic framework for interpreting non-linear nitrogen effects on bulb composition.

At the same time, pot experiments have clear boundaries. Absolute yield levels and nitrogen use efficiency under agronomically realistic fertilization regimes cannot be inferred directly from a controlled pot setup, and field environments introduce additional variation in soil properties, weather, and management. Therefore, the present results should be interpreted primarily as a physiological framework that identifies variety-dependent response patterns and thresholds, rather than as evidence for field-level optimization. Follow-up work under field conditions with practice-relevant N rates and timing (combined with targeted measurements of NUE components) will be important to evaluate how the observed metabolic trade-offs translate to agronomic performance and quality outcomes in real production systems.

## 5 Conclusion

This study demonstrates that onion bulb responses to nitrogen are strongly variety-dependent and become non-linear under oversupply. The landrace variety Birnenförmige and the hybrid variety Hytech F1 started from distinct carbon–nitrogen baselines, which shaped the timing and magnitude of metabolic reprogramming along the nitrogen gradient. Intermediate nitrogen levels supported bulb growth, whereas the highest nitrogen level shifted the system into an oversupply regime characterized by residual mineral N and a pronounced shift toward nitrogen-rich soluble composition. These changes were accompanied by clear quality trade-offs: phenolic antioxidants declined in parallel with higher nitrogen status and lower elemental C:N ratios, while putatively annotated sulfur-containing compounds increased even as pyruvate-based pungency dropped, suggesting a possible compositional decoupling within the flavor pathway. Together, the results provide an integrative physiological framework linking nitrogen status to coordinated changes in growth, primary metabolism, and key quality-related metabolite groups in onion bulbs.

## Data Availability

The original contributions presented in the study are included in the article/**Supplementary Material**. Further inquiries can be directed to the corresponding author.
