# Supplementary material for: Nitrogen supply modulates carbon–nitrogen balance and metabolic profiles in contrasting onion varieties
Source: Front Plant Sci. 2026 Aug 14;17:1886540. doi: 10.3389/fpls.2026.1886540 (PMC13521915; doi:10.3389/fpls.2026.1886540)
Supplement: Supplementary file 1 [file DataSheet1.pdf]

Nitrogen Supply Modulates Carbon–Nitrogen Balance and Metabolic Profiles in Contrasting Onion Varieties

M. L. Romo-Pérez*, C. H. Weinert[2], B. Egert[2], S. E. Kulling[2], C. Zörb[1]

[1] University of Hohenheim, Institute of Crop Science, Quality of Plant Products 340e, Schloss Westflügel, 70599 Stuttgart, Germany.

[2] Max Rubner-Institut, Institute for Foods of Plant Origin, Haid-und-Neu-Straße 9, 76131 Karlsruhe, Germany

[3] Max Rubner-Institut, Department of Safety and Quality of Fruit and Vegetables, Haid-und-Neu-Straße 9, 76131 Karlsruhe, Germany

Corresponding author

M.L. Romo-Pérez (m.romoperez@uni-hohenheim.de)

Keywords: *Hybrids, landraces, Allium cepa L., nitrogen, quality, carbon, metabolomics*

1. Untargeted GC×GC-MS metabolome analysis

Sample extraction, evaporation and derivatization

Work step	Action
	Extraction
1	Thaw study samples and QC sample aliquots
2	Add 750 µL of methanol
3	Add 20 µL of a mixture of internal standards containing hexylamine (5 mM), 1-*O*-methyl-2-desoxy-d-ribose (5 mM), 2-chlorophenylacetic acid (5 mM), *p*-chloro-l-phenylalanine (2 mM), 3-*O*-methyl-D –glucopyranose (2 mM), 5-bromo-2,4-dihydroxybenzoic acid (4 mM), and phenyl-β-D -glucopyranoside (1.25 mM) in methanol/water
4	Mix samples vigorously
5	Incubate samples for 10 min at 35 °C and 1,400 rpm in a shaker
6	Remove insoluble matter by a short centrifugation
7	Transfer 700 µL of the supernatants into new 2 mL reaction tubes
8	Re-extract the samples (repeat steps 2, 4, and 5)
9	Combine and mix both supernatants
10	Sediment insoluble matter by centrifugation for 5 min and 16100 × g
11	Transfer 30 µL of the combined supernatants to screw-capped glass vials with 300 µL micro inserts
	Evaporation
12	Dry samples in a rotary vacuum concentrator (RVC 2-25 CDplus/Alpha2-4 LSCbasic; Martin Christ GmbH, Osterode, Germany) for 50 min at 40 °C and p< 1 mbar
13	Re-dissolve samples again in 10 µL of methanol
14	Dry samples again to remove traces of water (30 min, 40 °C, p< 1 mbar)
	Derivatization
15	Add 20 µL of methoxylamine-hydrochloride in dried pyridine (20 mg/mL)
16	Incubate for 1 h at 40 °C and 1,000 rpm in a shaker
17	Add 50 µL of MSTFA
18	Incubate for 1 h at 65 °C and 800 rpm in a shaker
19	Add 10 µL of retention index marker mixtures (n-alkanes, FAMEs) only to daily RI-QC sample

GC×GC-MS system

Instrument component	Name	Manufacturer
Gas chromatograph (single oven)	GC-2010	Shimadzu Corp, Kyoto, Japan
Single quadrupole mass spectrometer	QP2010 Ultra	Shimadzu Corp, Kyoto, Japan
Autosampler	PAL3 RTC	CTC Analytics, Zwingen, Switzerland
PTV Injector	OPTIC-4	GL Sciences, Eindhoven, The Netherlands
Loop-type cryogenic modulator	ZX2	ZOEX Corp., Houston, USA
Roughing pump	RV-3	Edwards, Crawley, UK

GC×GC method and consumables

Carrier gas	Helium 5.0
GC mode	Constant velocity
Purge flow	3 mL/min
Initial column head pressure	144.8 kPa
Liner type	Deactivated, fritted split liner with quartz wool
Primary column	Rxi-5SilMS, ^{1}L = 30 m plus 7 m of an integrated pre-column, $^{1}d_c$ = 0.25 mm, $^{1}d_f$ = 0.25 µm (Restek, Bellefont, USA)
Secondary column	BPX50, $^{2}L_{total}$ = 2.0 m, including a "separation segment" of $^{2}L_{sep}$ = 0.7 m, $^{2}d_c$ = 0.15 mm, $^{2}d_f$ = 0.15 µm (Trajan Scientific, Ringwood Victoria, Australia)
GC temperature ramp	90 °C → 3.5 °C/min → 280 °C→ 5 °C/min → 320 °C → 10 °C/min → 340 °C (hold 5.71 min)
Run time	70 min
Injection mode	Cold split
PTV temperature program	90 °C → 60 °C/s → 200 °C (hold 1 min) → 60 °C/s→ 280 °C (hold until end of run)
Split ratio program	1:2 (hold 1 min) → 1:50 (hold 4 min) → 1:10 (hold 25 min) → 1:2 (hold until end of run)
Injection volume	1.0 µL
Injector syringe	10 µL, with PTFE-tipped plunger (Trajan Scientific, Ringwood Victoria, Australia)
Syringe wash solvent 1	Acetone
Syringe wash solvent 2	Heptane

Modulator settings

Modulation period (PM)	2.2 s
Cold jet temperature	-90 °C
Hot jet temperature program	200 °C (hold until 15 min) → 250 °C (hold until 30 min) → 300 °C (hold until 45 min) → 380 °C (hold until 68 min) → 200 °C (hold until end of run)
Hot jet pulse duration	350 ms

MS settings

Interface temperature	290 °C
Ion source temperature	250 °C
Ionization mode	EI (70 eV)
MS Mode	Scan
Scan speed	20.000 u/s
Scan range	m/z 60-550
Event time	30 ms
Data acquisition frequency	33 s^{-1}
Data acquisition period	4.8 -70 min
Detector voltage	Between 0.88 – 0.97 kV, depending on chromatogram window and measurement week

1. Results

Table S1 Two-way ANOVA results for morphological and quality traits. P-values are shown for the main effects of variety and nitrogen treatment and for the variety × nitrogen interaction. Formal interpretation of variety-specific nitrogen responses was based on the significance of the variety × nitrogen interaction.

Trait	Variety	Nitrogen	Variety × nitrogen	Formal interpretation
**Average bulb weight per pot**	0.535	0.013	0.139	Significant main effect of nitrogen; no significant interaction
**Bulb diameter**	<0.001	<0.001	0.080	Significant main effects of variety and nitrogen; no significant interaction
**Bulb height**	<0.001	<0.001	0.543	Significant main effects of variety and nitrogen; no significant interaction
**Leaf number**	<0.001	0.001	0.013	Significant variety × nitrogen interaction; interpret nitrogen effects within each variety
**Firmness**	<0.001	0.002	0.249	Significant main effects of variety and nitrogen; no significant interaction
**Dry matter**	<0.001	0.009	0.176	Significant main effects of variety and nitrogen; no significant interaction
**Total soluble solids**	<0.001	0.053	**0.051**	Significant main effect of variety only
**Pyruvate**	0.011	<0.001	0.375	Significant main effects of variety and nitrogen; no significant interaction
**Antioxidant capacity**	<0.001	<0.001	**0.006**	Significant variety × nitrogen interaction; interpret nitrogen effects within each variety

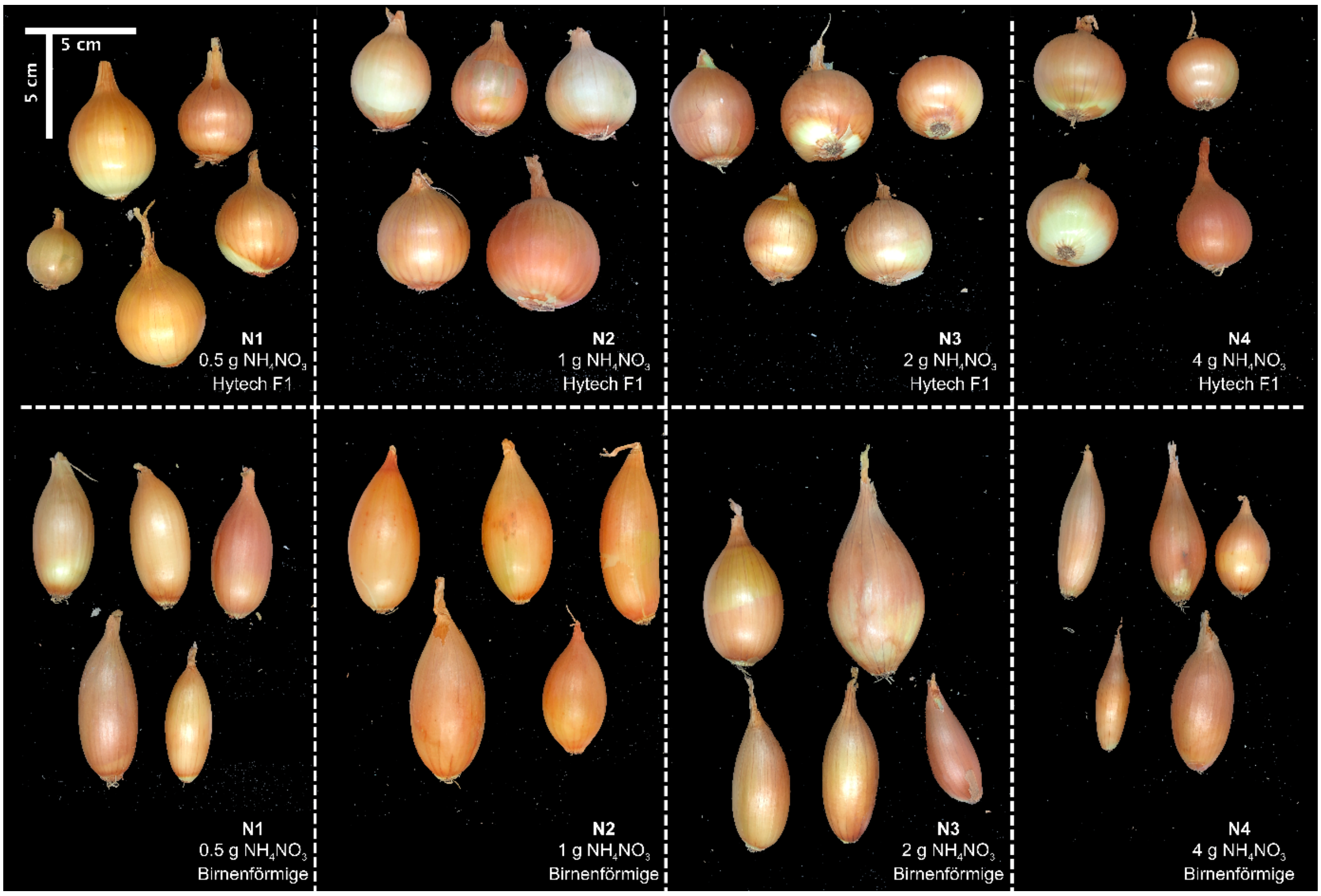

Fig. S 1 Representative bulbs of the two onion varieties grown under four nitrogen levels (N1–N4). Hytech F1 formed round bulbs that decreased in size at higher N supply, while Birnenförmige maintained its elongated shape up to N3, with visible size reduction only at N4. The visual patterns correspond to the quantitative differences in bulb weight, height, and diameter shown in Figure 2A–C.

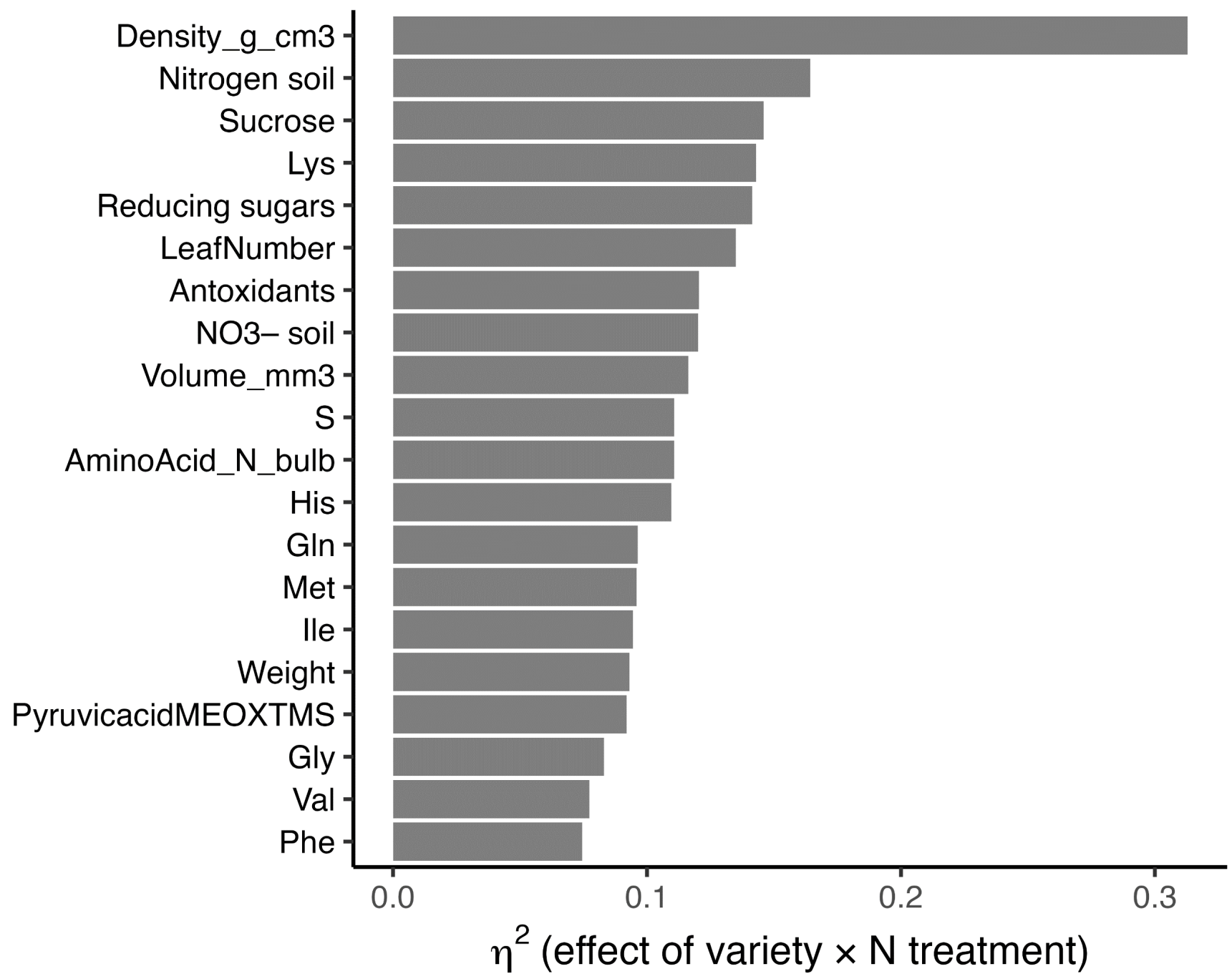

Fig. S 2 Top-ranked traits affected by the variety × nitrogen interaction (η^2).

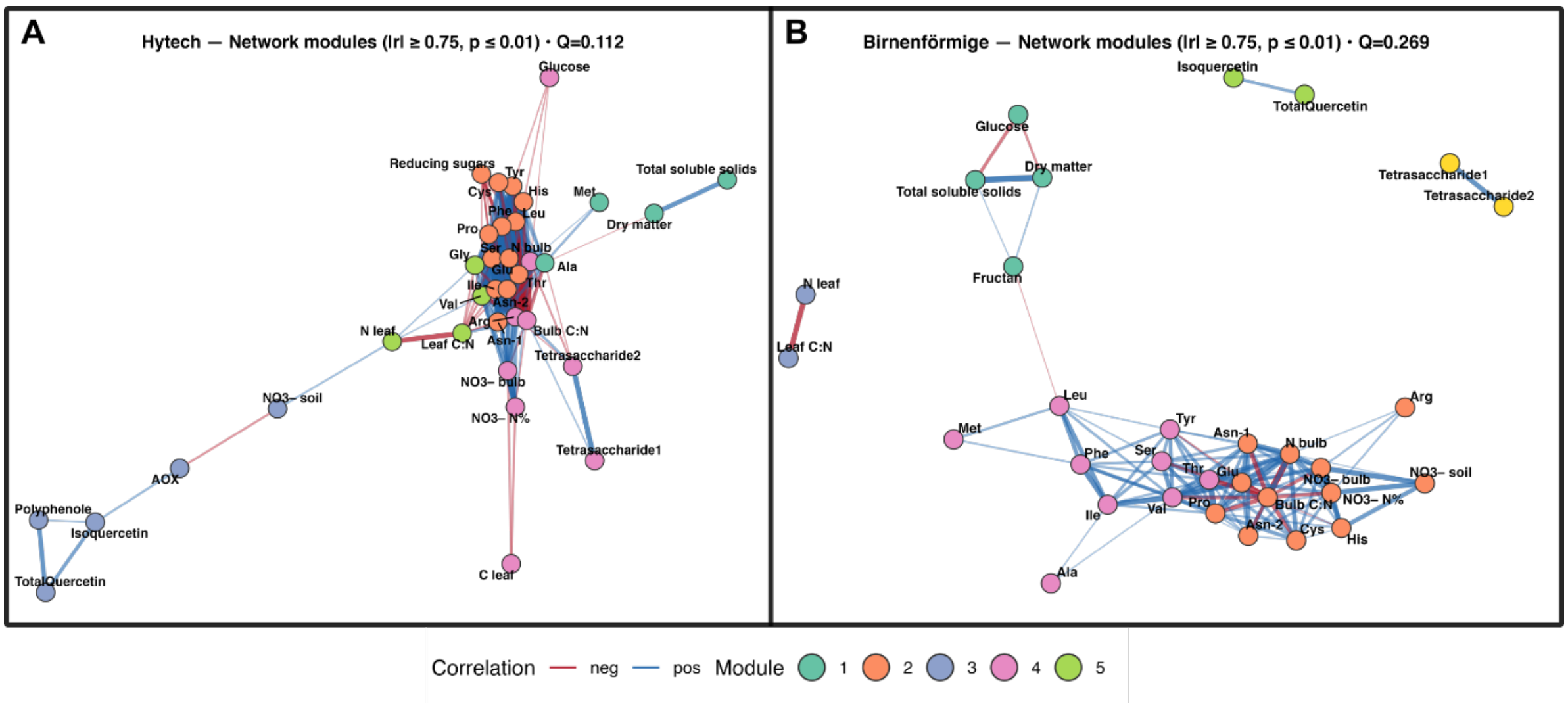

Fig. S 3 Variety-specific metabolite correlation networks based on Pearson correlations across all nitrogen treatments. Nodes represent individual metabolites and are coloured by Louvain module; edges denote significant correlations (|r| ≥ 0.75, p ≤ 0.01; blue = positive, red = negative). Networks are shown separately for Hytech (left) and Birnenförmige (right); modularity values (Q) indicate the strength of the module structure.
